# Coexisting giant splenic artery aneurysm and non-functioning pancreatic neuroendocrine tumor

**DOI:** 10.12669/pjms.36.4.1869

**Published:** 2020

**Authors:** Omer Yalkin, Mustafa Yener Uzunoglu, Fatih Altintoprak, Ali Muhtaroglu

**Affiliations:** 1 Omer Yalkin Department of Surgical Oncology, Bursa City Hospital, Bursa, Turkey; 2 Mustafa Yener Uzunoğlu Department of General Surgery, Sakarya Teaching and Research Hospital, Sakarya, Turkey; 3 Fatih Altıntoprak, Department of General Surgery, Sakarya University, Faculty of Medicine, Sakarya, Turkey; 4 Ali Muhtaroglu, Department of General Surgery, Sakarya University, Faculty of Medicine, Sakarya, Turkey

**Keywords:** Neuroendocrine Tumors, Aneurysm, Splenic Artery

## Abstract

The splenic artery aneurysm (SAA) is rare clinical entity which is the third most common intra-abdominal aneurysm. Pancreatic neuroendocrine tumors (pNETs) are rare malignancies which comprise less than 2% of all pancreatic tumors. Non-functioning pancreatic neuroendocrine tumors set forth up to 90% of all PNETs. Sixty-seven-year-old female presented to our polyclinic with increasing pain in the left upper quadrant in the previous three months. A computed tomographic angiography revealed 13x13x12 cm sized regular bounded aneurysmatic expansion of medium part of splenic artery. In addition there was a 8x7 mm sized hypoecoic lesion in the distal pancreatic tissue. Distal pancreatectomy, splenic aneurysm resection and splenectomy was performed. Pathological results revealed that there was a 12 cm sized giant true splenic aneurysm and 0.7 cm sized neuroendocrine tumor in the pancreatic tissue. This manuscript is presentation of surgical approach to a case with coexistence of these two rare conditions.

## INTRODUCTION

SAA is very rare, but has been diagnosed more often with the development of imaging possibilities. Because the risk of rupture, SAA is one of the most dangerous treatable surgical diseases and with mortality in the range of 10 to 40%.^1^ Over the last decades, pancreatic neuroendocrine tumors (pNETs), particularly non-functioning (NF) ones, have been identified with increasing frequency like SAAs.^2^ NF-pNETs have a wide spectrum of clinical behaviors, ranging from a benign nature, to the malignant lesion with extensive metastase.^3^ This paper reports the coexistence of these two rare conditions.

## CASE REPORT

**Fig.1:**
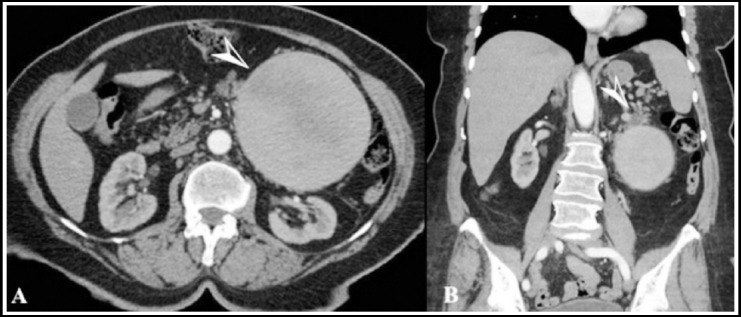
A computed tomographic (CT) angiogram; arrow head showed an aneurysm arising from the middle portion of splenic artery and arrow head showed an hypoechoic lesion arising from the distal pancreas.

**Fig.2:**
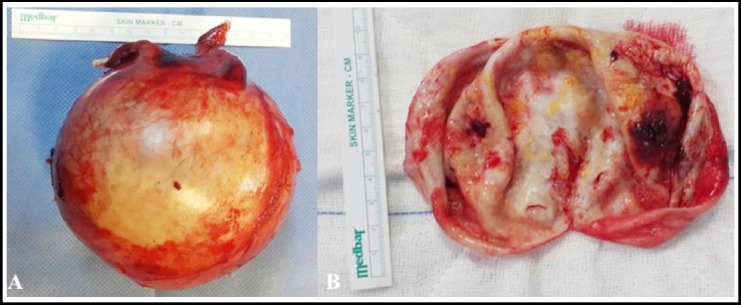
Intraoperative image of the specimen after removal, showing the aneurysm.

A 67-year-old multiparous (gravida 4, parity 3) female presented to our polyclinic with increasing pain in the left upper quadrant in the previous three months. She had no history of hypertension, trauma, aneurysm, or pancreatitis. In physical examination, she had a swelling in her left upper quadrant. All laboratory tests were within the normal range. A computed tomographic angiography revealed 13x13x12 cm sized regular bounded aneurysmatic expansion of medium part of splenic artery. In addition there was an 8x7 mm sized hypoecoic lesion in the distal pancreatic tissue (Fig.1). Plasma chromogranin A, plasma neuron-specific enolase, synaptophysin, pancreatic polypeptide, Cancer Antigen 19-9 tests was performed. The level of these markers were normal. The patient was scheduled for robotic surgery. During the operation, the portion of the splenic artery proximal to the SAA was dissected and looped with the da Vinci S Surgical System (Intuitive Surgical Inc, Sunnyvale, CA). Because of techniqual problems, operation was performed with open surgery. Distal pancreatectomy, splenic aneurysm resection and splenectomy was performed (Fig.2). The patient’s postoperative course was uneventful and he was discharged on the third postoperative day. Pathological results revealed that there was a 12 cm sized giant true splenic aneurysm and 0.7 cm sized neuroendocrine tumor in the pancreatic tissue. The patient was directed to the medical oncology clinic.

## DISCUSSION

SAAs are a rare entity commonly diagnosed incidentally. The true incidence of SAA is not clear, studies of autopsies, angiography findings showed rates between 0.01% and 10.4%, respectively. Most SAA are located in the distal portion of the artery, close to splenic hilum.^4^

Portal hypertension, cirrhosis and liver transplantation are also associated with SAA. Less common etiologies are alpha 1-antitrypsin deficiency, inflammatory and infectious disorders. Arteriosclerosis is probably a secondary process occurring from primary degeneration of the media. Pseudoaneurysms are caused by pancreatitis, trauma and may also be iatrogenic.^5^

There is no documented algorithm regarding the approach of treating SAA based on prospective randomized trials; treatment is planned on a case by case basis. It seems to attempt transcatheter embolization or endovascular techniques in patients with serious co-morbidities and favorable vascular anatomy. If this fails, laparoscopic treatment should be the next option.^6^

Pancreatic neuroendocrine neoplasms (pNENs), originating from diffuse neuroendocrine cells. It’s include well differentiated pancreatic neuroendocrine tumors (pNETs) and neuroendocrine carcinomas (pNECs). pNENs comprise only 1% to 2% of all pancreatic neoplasms and about 7–9% of all gastroenteropancreaticneuroendocrine tumors (GapNETs).^7^

Depending on the hormone produced, different clinical manifestations arise. Conversely most pNEN’s are asymptomatic due to lack of hormone secretion. As a result, the majority of patients are diagnosed at an advanced stage and they produce either non-specific symptoms or mass-related symptoms, the latter depending on the location and size of the lesion.^8^

Diagnostic point of pNEN is laboratory tests which confirm the oversecretion of the specific pancreatic hormone should be performed (Insulin, Glucagon, Somatostatin, Vasoactive intestinal peptide, Pancreatic polypeptide, Neurotensin, Ghrelin). Chromogranin A (CgA), pancreatic polypeptide (PP), and pancreastatin has a diagnostic and prognostic role for both functioning and non-functioning tumors.^8^

Various imaging modalities including computed tomography (CT), magnetic resonance imaging (MRI), somatostatin receptor scintigraphy (OctreoScan®), positron emission tomography (PET), endoscopic ultrasonography (EUS), and selective angiography.^9^

Surgical resection with regional lymph node dissection is curative treatment option and is recommended to all patients with early-stage well-differentiated pNENs. Advanced cases are incurable and systemic therapies form the mainstay. Somatostatin analogues are effective for controlling the hormone-excess state in functional pNENs.^10^

## CONCLUSION

The coexistence of giant splenic artery aneurysm and non-functioning pancreatic neuroendocrine tumor is very rare. We believe that a detailed radiological examination is very important for detecting accompanying anatomic abnormalities when surgical intervention is planned.

### Authors’ Contribution

**OY & AM:** Conceived, designed & editing of manuscript. and **Omer Yalkin** is responsible and accountable for the accuracy or integrity of the work.

**MY:** Did data collection and manuscript writing.

**FA:** Did review and final approval of manuscript.
